# Basement membrane dynamics and mechanics in tissue morphogenesis

**DOI:** 10.1242/bio.059980

**Published:** 2023-08-02

**Authors:** Uwe Töpfer

**Affiliations:** Department of Cellular and Physiological Sciences, University of British Columbia, Vancouver, Canada, V6T 1Z3

**Keywords:** Extracellular matrix, Laminin, Collagen, Nidogen, Perlecan

## Abstract

The basement membrane (BM) is a thin, planar-organized extracellular matrix that underlies epithelia and surrounds most organs. During development, the BM is highly dynamic and simultaneously provides mechanical properties that stabilize tissue structure and shape organs. Moreover, it is important for cell polarity, cell migration, and cell signaling. Thereby BM diverges regarding molecular composition, structure, and modes of assembly. Different BM organization leads to various physical features. The mechanisms that regulate BM composition and structure and how this affects mechanical properties are not fully understood. Recent studies show that precise control of BM deposition or degradation can result in BMs with locally different protein densities, compositions, thicknesses, or polarization. Such heterogeneous matrices can induce temporospatial force anisotropy and enable tissue sculpting. In this Review, I address recent findings that provide new perspectives on the role of the BM in morphogenesis.

## Teaser

**Summary:** Basement membranes (BMs) are critical for morphogenesis. This Review provides an overview of the mechanisms by which BMs can be remodelled to regulate the shape of tissues and organs.

## Introduction

The basement membrane (BM) is a sheet-like matrix surrounding most tissues and organs in the extracellular space. This structure has a profound role in tissue morphogenesis and maintenance and is important for cellular behavior like cell migration and cell signaling (Sherwood, 2021; Yurchenco, 2011). Moreover, dysregulation of BM homeostasis has been associated with a series of human diseases, including fibrosis and cancer (Sekiguchi and Yamada, 2018).

**Fig. 1.**
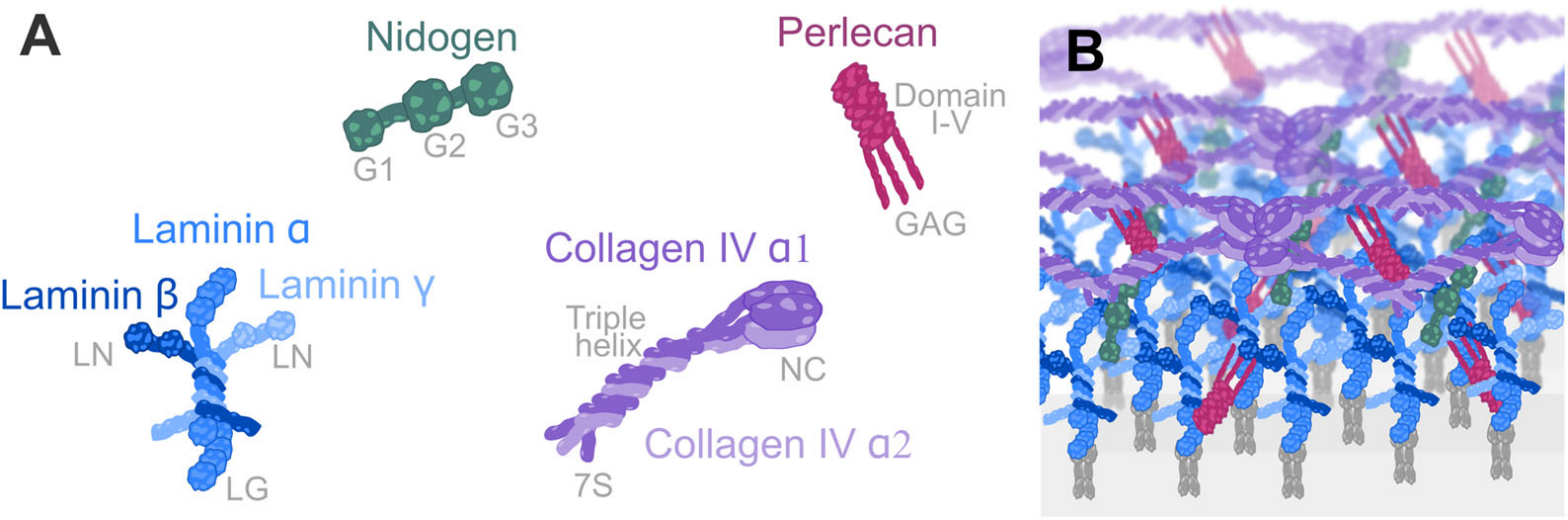
**Protein structure of BM components and composition.** (A) Laminin (blue) is a coiled-coil heterotrimer interacting with its globular domains (LG) to cell-surface receptors and with its terminal domain (LN) at its short arms with other Laminin heterotrimers. Laminin comprises one α-, one ß, and one γ-subunit. Nidogen (green) consists of three globular domains (G1, G2, G3). Perlecan (red) has glycosaminoglycan (GAG) chains and the domains I-V. Collagen IV (violet) is a triple-helix building heterotrimer composed of either two α1- and one α2-subunit with 7S and a non-collagenous (NC) domain at their termini. (B) An exemplary composition of a planar organized BM. Laminin (blue) builds an internal network at the cell surface and interacts with cell-surface receptors, like integrins (grey). Collagen IV (violet) builds an outer network. Both networks are linked through Nidogen (green) and Perlecan (red).

The BM can consist of hundreds of distinct proteins; however, across metazoan life, most BMs entail the components Laminin, Collagen IV, Nidogen, and Perlecan (Hynes, 2012; Jayadev et al., 2022). The glycoprotein Laminin builds a coiled-coil heterotrimer, composed of an α-, β- and γ-subunit each (Fig. 1; Bruch et al., 1989; Engel et al., 1981). After secretion into the extracellular space, Laminins interact with their LG domain to cell surface receptors like Integrin. In contrast, their LN domains build ternary nodes by the interaction of either a one α-, one β- and one γ-subunit of a different Laminin heterotrimer (Fig. 1; Ettner et al., 1998; Yurchenco and Cheng, 1993). Collagen IV, a hetero-trimeric protein with a triple helical collagen domain composed of Gly-X-Y repeats and with cysteine-rich NC1 and 7S domains at their termini, assembles with interactions of these terminal domains into a network (Fig. 1; Timpl et al., 1981). Both networks are crosslinked with proteins like the glycoprotein Nidogen (also known as Entactin) and the proteoglycan Perlecan (Fig. 1; Aumailley et al., 1989; Fox et al., 1991; Hopf et al., 2001; Reinhardt et al., 1993). Moreover, the BM can contain components like Agrin, Fibulin, Sparc, and Fibronectin in vertebrates, Glypicans, Collagen XV/XVIII, and more. In addition, single components can be cleaved or truncated by extracellular matrix metalloproteases like Mmp (Matrix metalloproteases) or AdamTS (a disintegrin metalloprotease with thrombospondin motif) gene family member (Table 1; Kelwick et al., 2015; Page-McCaw et al., 2007), resulting in highly complex and dynamic structures. The BM complexity is even higher in Vertebrates than in Invertebrates (Table 1). Model organisms like *Drosophila melanogaster* and *Caenorhabditis elegans* have smaller genomes than humans with fewer genes encoding for BM components. Nevertheless, the genes and biological processes are very similar in model organisms and humans. Moreover, the usage of genetic model organisms is cost-efficient as they reproduce and develop fast and can be bred without much space in great numbers. Thus, studies on genetic model organisms are critical to understanding the function of this protein meshwork and uncovering the mechanisms of how BMs are required for tissue and organ morphogenesis.

**Table 1.**
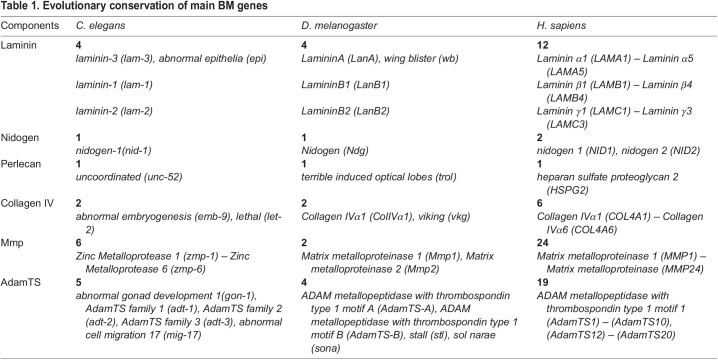
Evolutionary conservation of main BM genes

The formation of tissues and organs during development depends on generating mechanical force. Force transmission through cell-receptor adhesion and force generation through the actomyosin networks drive morphogenesis (Heisenberg and Bellaïche, 2013; Molnar and Labouesse, 2021). The BM provides a scaffold for cells to attach to and interact with and can affect cell mechanics through its physical properties. Stiffness, elasticity, viscoelasticity, and topography of the BM can affect how cells adhere to and migrate through it, which can create tension on the cells (Elosegui-Artola, 2021; Paci and Mao, 2021). Additionally, the BM contains many signaling molecules, such as growth factors and cytokines, that can activate cell signaling pathways and influence their behavior. The BM can indirectly affect cell mechanics through a complex interplay of mechanical, biochemical, and protein-protein interactions. By regulating these forces, the BM fulfills various functions for morphogenesis. BMs provide cells a substratum for adhesion, are required for cell polarization and cell migration, proliferation, act as a barrier, and as a reservoir for morphogens, all described in excellent reviews elsewhere (Sekiguchi and Yamada, 2018; Sherwood, 2021; Yurchenco, 2011).

In this Review, I will provide an overview of recent work addressing how the mechanical properties of the BM regulate tissue and organ shape. To do so, I will describe the current model of BM assembly and discuss interdependencies for correct BM maintenance. Second, I will address new findings in BM turnover and remodeling. Third, I will discuss the role of mechanical heterogeneity forming complex structures during morphogenesis, pointing to the close linkage between composition, mechanics, and tissue shape. Finally, I will declare the future perspectives and how methodological advances in genetics, imaging, and techniques measuring direct biophysical properties can be used to characterize better the role of BM dynamics and mechanics for morphogenesis.

## BM assembly, hierarchy, and dependencies

**Fig. 2.**
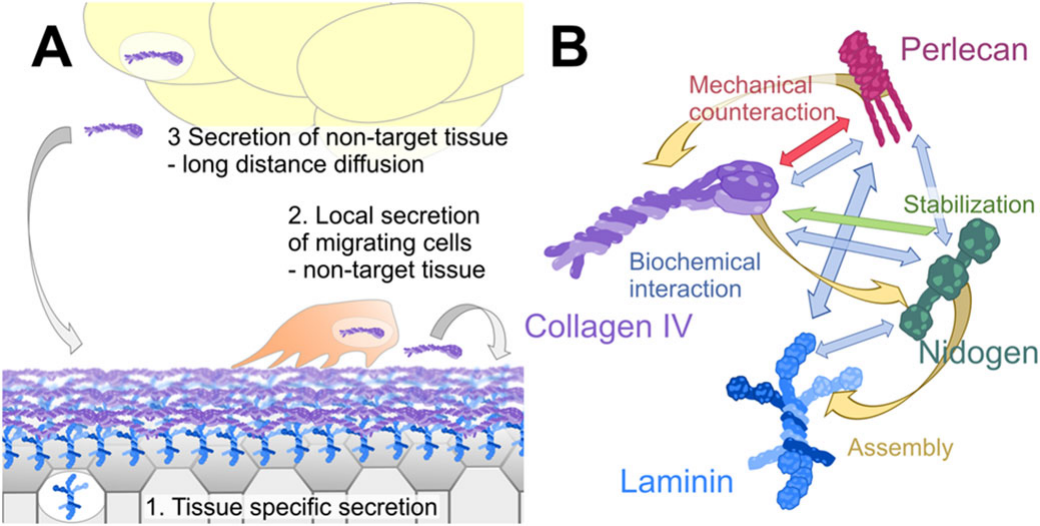
**BM secretion, assembly, and protein interactions.** (A) Secretion of BM components into the extracellular space can be organized through (1) tissue-specific expression, local secretion of cells, (2) migrating over the target tissue, and (3) secretion by non-target tissues with subsequent long-distance diffusion. (B) Assembly (yellow arrows) of BM components needs a strict hierarchical order starting with Laminins (blue), which interact with cell-surface receptors. Nidogen (green) requires the presence of Laminin to assemble into the network. Collagen IV (violet) assembles thereafter. Perlecan (red) needs Collagen IV for assembly. Biochemically, Nidogen and Perlecan interact with all other components, while Laminin and Collagen IV only bind to Nidogen and Perlecan, respectively (blue arrows). Nidogen seems to have a unique role in stabilizing the BM, maybe through regulating Collagen IV turnover (green arrow). Perlecan and Collagen IV mechanically counteract (red arrow).

The proper functionality of the BM depends on its correct maturation. *Ex vivo* experiments have shown that isolated Laminin in a solution can aggregate and assemble in a matrix by itself (Yurchenco et al., 1985). The question arises of whether a BM could build itself *in vivo* if enough ‘material’ is in the extracellular space. Since the BM is a scaffold with an inner and an outer network and proteins in between, one would expect a temporal or a spatial organization of gene expression or both. Notably, in contrast to cultured cells, the origin of synthesis can differ *in vivo.* The final destination of an extracellular matrix (ECM) protein can be organized via local expression by the tissue itself or local secretion of migrating cells, such as the secretion of non-target tissues and capture to the corresponding tissue (Fig. 2A; Brown, 2011). Three findings support the idea of a temporal-spatial BM assembly. First, a recent publication has quantified protein level dynamics of the GFP-tagged version of the main components over embryonic development in *Drosophila* (Matsubayashi et al., 2017). Thereby, Laminin A (one of two α-subunits) is expressed first, followed by Collagen IV, and a final peak of Perlecan signal reveals a temporal hierarchical deposition (Fig. 2B, yellow arrows). Second, in *Drosophila* and mice expression and gene regulation analysis indicate a tissue-specific expression of Laminin (Töpfer et al., 2019; Tunggal et al., 2000; Wolfstetter and Holz, 2012), while Collagen IV is mainly secreted by non-target tissues (Gupta et al., 1997; Pastor-Pareja and Xu, 2011; Simon-Assmann et al., 1990). These modes of secretion may indicate an initial assembly of the inner Laminin network by tissues themselves followed by more or less unspecific deposition and incorporation of the overlaying Collagen IV network (Fig. 2A). The third indication that gives evidence for a requirement of a controlled BM assembly is provided by the protein dependency analyzed in mutant conditions and described below.

The loss of Laminin in the *Drosophila* embryo leads to unspecific accumulations of other BM main components coupled with several defects during morphogenesis in the nervous system, somatic muscles, heart, gut, and trachea (Urbano et al., 2009; Wolfstetter and Holz, 2012). Mutant mice lacking Collagen IV still show Laminin and Nidogen in a BM-like matrix, associated with lethality due to loss of BM stability (Poschl et al., 2004). This suggests a major role of Laminin for initial BM assembly, while Collagen IV provides the BM with mechanical strength. Notably, in some examples, like the *C. elegans* pharyngeal BM (Jayadev et al., 2019), during wound repair of the *Drosophila* epidermis (Ramos-Lewis et al., 2018) or after initial BM assembly in the *Drosophila* egg chamber (Töpfer et al., 2022a) Collagen IV assembly is Laminin-independent.

A long-lasting question in matrix biology is how the two networks are interconnected. For a long time, Nidogen has been suggested as an essential component to recruiting Collagen IV and Perlecan due to its ability to bind both networks with distinct domains (Fig. 2B, blue arrows; Aumailley et al., 1993; Ho et al., 2008; Mayer et al., 1995). Nidogen protein consists of three globular domains (G1 to G3). Essential for incorporation is the interaction of the G3 domain with the Laminin γ-subunit, while the G2 domain allows linkage to Perlecan and Collagen IV (Fox et al., 1991; Hopf et al., 2001; Mann et al., 1989; Reinhardt et al., 1993). Studies in *Drosophila*, *C. elegans*, and mice have shown Nidogen is dispensable for assembly of the other core components (Bader et al., 2005; Dai et al., 2018; Kang and Kramer, 2000; Töpfer and Holz, 2020; Wolfstetter et al., 2019). Notably, all studies show a role of Nidogen in BM stability and tissue integrity (Fig. 2B, green arrow). However, the question of Laminin and Collagen IV network linkage remains. Perlecan can bind both networks, too (Behrens et al., 2012; Costell et al., 1999), and there could be a redundant function between Nidogen and Perlecan or other additional linker proteins.

One particular protein interaction has been described to be essential for morphogenesis. Perlecan and Collagen IV seem to have a counteracting function in sculpting tissues (Fig. 2B, red arrow). For example, in the *Drosophila* wing disc, while Perlecan assembly into the BM depends on Collagen IV, once present, it counters the constricting abilities of Collagen IV (Fig. 2B). Without Collagen IV, the normally columnar epithelial cells show a flattened phenotype. In contrast, the knockdown of Perlecan results in a super-constricted wing disc (Bonche et al., 2022; Pastor-Pareja and Xu, 2011). This mutual dependency has also been described in the *C. elegans* neuromuscular junction, where Perlecan promotes growth of ectopic presynaptic buttons and invasion into the nonsynaptic region, and the gonad, in which loss of Perlecan results in progressive compaction (Jayadev et al., 2022; Qin et al., 2014). Further examples in *Drosophila* are the condensation of the ventral nerve cord (VNC), here Perlecan is required to keep the tissues structural integrity and the elongation of the *Drosophila* egg chamber, but here, like for Collagen IV knockdown, knockdown of Perlecan leads to an inhibition of elongation, too (Isabella and Horne-Badovinac, 2015; Pastor-Pareja and Xu, 2011; Skeath et al., 2017; Töpfer et al., 2022a). Thus, Perlecan seems to have a general function in sculpting tissues by maintaining the BM structural integrity (Jayadev et al., 2022). In particular, Perlecan's mechanical function must be carefully analyzed due to its multiple roles in regulating cell signaling events (Whitelock et al., 2008). In summary, correct assembling of the BM components is required for morphogenesis and tissue integrity.

## Turnover and remodelling – dynamics of BM proteins

The amount of each single ECM component assembled into the BM influences the composition and the biophysical and biochemical aspects. Hence, BM homeostasis is biased to the turnover of single ECM proteins. Turnover is the total change in protein amount over time and reflects the rate of synthesis and degradation (Ross et al., 2021). Incorporating newly synthesized material requires recruitment through available interaction partners (Dörrbaum et al., 2018). The degradation of ECM proteins is mediated by metalloproteases and lysosomal enzymes (Cawston and Young, 2010; Machado et al., 2023; Page-McCaw et al., 2007). The origin of synthesis for each component of the BM, the complex protein interactions, temporospatial expression, and substrate specificity of matrix-degrading proteases are crucial factors in tuning BM turnover.

The BM has been shown to stabilize tissues during embryonic development (Poschl et al., 2004). Consistent with the stabilizing role of the BM, studies in adult mice show a half-life of BM components of weeks (Decaris et al., 2014; Trier et al., 1990). Two recent studies show now a surprisingly high turnover of BM components. In *Drosophila* embryogenesis, Matsubayashi et al. (2020) measured the turnover of Collagen IV and Perlecan. The authors measured a half-life of these proteins of ∼7-10 h using mathematical modeling of the BM levels to homeostasis and by using pulse-chase experiments with ectopically expressed Collagen IV subunit tagged with photoconvertible fluorophores. Moreover, they identified two proteins involved in Collagen IV turnover. In *Mmp1* (*Matrix metalloprotease 1*) mutants, they observed a ∼20% decrease in turnover and found the opposite effect with an ∼20% increase in turnover in *Nidogen* mutants. This suggests that Mmp1 is involved in Collagen IV degradation during embryogenesis but is not the only factor. Nidogen stabilizes Collagen IV, consistent with its previously suggested role in BM and tissue stabilization (see above). As a consequence of disturbed Collagen IV turnover, tissue morphogenesis is disturbed through a slowed-down condensation of the VNC (Matsubayashi et al., 2020). Here, the GFP signal of whole embryos was used, which provides mean values of all tissues. A future question remains to be solved. It is currently unknown whether different tissues provide distinct turnover rates in this model.

In *C. elegans*, Keeley et al. (2020) tagged 29 BM components and receptors endogenously with mNeon-Green and quantified BM dynamics. Consistent with the idea of a regulated BM assembly, Laminin subunits and Nidogen are expressed before Collagen IV and further specification. Interestingly, *Papilin* (*Ppn*) an AdamTS-like gene (a disintegrin metalloprotease with thrombospondin motif-like) is expressed during early development, too. Moreover, the authors show that this gene regulates Collagen IV turnover by promoting its removal, potentially via regulating the function of AdamTS proteases or the Collagen IV crosslinking by Peroxidasin. Like in the *Drosophila* embryo, turnover measurements using ‘fluorescence recovery after photobleaching’ (FRAP) analysis shows a rapid turnover in a few hours. Remarkably, Laminin and Collagen show a slower turnover than linker proteins like Nidogen or Fibulin. Another fascinating result of this study is the finding that Nidogen, Agrin, or Fibulin, move fast in the lateral direction, while the major scaffolding proteins Laminin and Collagen IV are more stable. This rebuts the idea of a fully stable structure and provides a new perspective on the BM in which Laminin and Collagen IV build a scaffold, and other components move in between.

This new perspective and more examples of BM component movement have been described recently in a review (Matsubayashi, 2022).

## Mechanical heterogeneity in tissue sculpting

How is the BM involved in shaping tissues and organs? Several mechanisms have been reported showing that a local BM remodeling is required for controlled outgrowth. In the following, I would like to point to some studied model tissues and compare the mechanisms of how the BM is remodeled in particular.

### Egg chamber elongation depends on a BM stiffness gradient

**Fig. 3.**
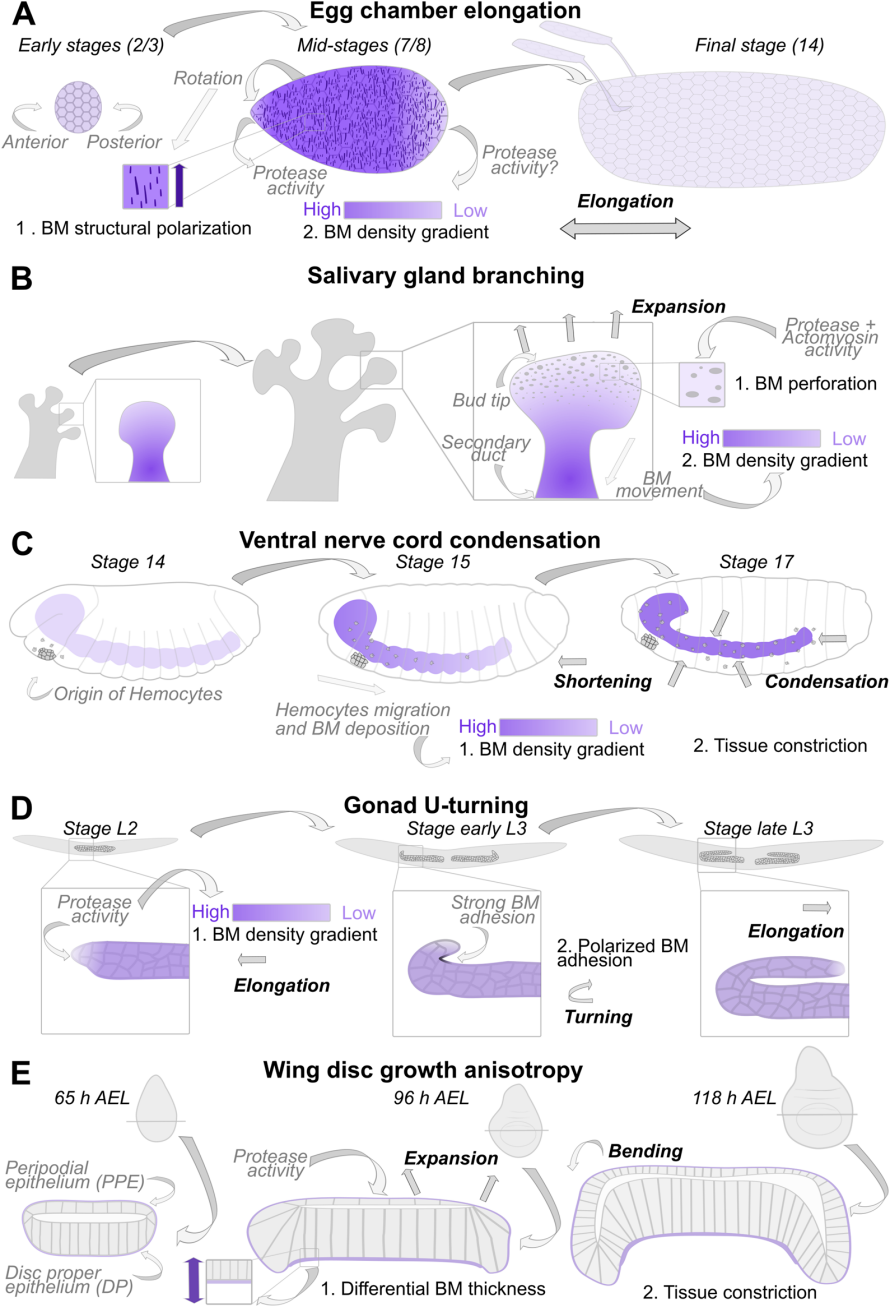
**Mechanisms of BM-dependent organ sculpting.** (A) *Drosophila* egg chamber elongation. In the early stages, egg chambers show a round morphology with a homogenous BM. Due to egg chamber rotation, the BM becomes polarized with fiber-like structures orientated perpendicular to the anterior-posterior axis (1). Additionally, in mid-stages of egg chamber development, the BM becomes heterogeneous, with a higher density in the central region and a lower in the terminal regions (2). This heterogeneity is associated with matrix protease expression at the anterior pole and has been assumed to be affected by protease expression at the posterior pole. Both aspects play a role in egg chamber elongation. (B) During branching morphogenesis of the mouse salivary gland, the BM becomes perforated at the tip of the bud (1) and moves toward the secondary duct, where it accumulates, leading to an increased density (2). The perforation of the BM depends on protease activity and increased actomyosin contractility. (C) The *Drosophila* ventral nerve cord (VNC) sculpting. The hemocytes originate in a cell population at the anterior of the embryo and migrate over tissues while deposing BM components. The transient higher level at the anterior (1) leads to a shortening of the VNC. As the BM is fully matured, it is required for constriction and condensation of the tissue (2). (D) During *C. elegans* gonad morphogenesis, protease activity at the tip cells leads to local BM degradation (1), stress release, and elongation along the anterior-posterior axis. Polarized BM adhesion at the tip of the tissue leads to a U-turn movement (2), followed by elongation towards the opposite direction. (E) The *Drosophila* wing disc has two epithelial layers, the disc proper epithelium (DP) and the peripodial epithelium (PPE) enclosed by a common BM. While the BM at the PPE is constantly remodeled by matrix protease expression and allows to complain the growth stress, leading to tissue expansion in this direction, the BM at the DP is thicker due to slow protease remodeling and resists growth. This leads to a bending of the tissue.

The *Drosophila* egg chamber is one of the most fascinating model tissues to study the role of the BM in organ formation. Since Haigo and Bilder, 2011 published ‘the global tissue revolutions’, researchers have studied how the BM forms the initial round egg chamber into an elongated organ. As a result of egg chamber rotation, the follicle cell-BM shows a polarization perpendicular to the anterior-posterior axis (Fig. 3A). Thereby, BM components are basolaterally secreted by the follicle cells during collective cell movement and assemble into the BM as fiber-like structures (Gutzeit et al., 1991; Haigo and Bilder, 2011; Isabella and Horne-Badovinac, 2016; Lerner et al., 2013; Zajac and Horne-Badovinac, 2022). This led to the global alignment of basal actin stress fibers (Cetera et al., 2014). A recent study indicates that these accumulations of proteins lead to an enhancement of BM stiffness (Chlasta et al., 2017). Whether these fiber-like structures and their orientation are indeed required to drive global BM mechanics remains an open question.

Additionally, during the main elongation phase (stages 5-8), a stiffness gradient with a higher stiffness in the central and lower stiffness in the terminal region is established. It seems to be essential for egg elongation (Crest et al., 2017). This stiffness gradient is tunable by altering Collagen IV secretion, disturbing BM assembly or composition, repealing the planar cell polarity, and inhibiting the JAK/Stat signaling pathway (Chlasta et al., 2017; Crest et al., 2017; Díaz de la Loza et al., 2017; Töpfer et al., 2022a). The resulting mechanical heterogeneity is required to control cellular behavior essential for egg elongation. Local alteration of junctional modeling, regulated by an Src tyrosine kinase, leads to the reorientation of the follicle cells in the anterior part (Chen et al., 2019). A recent study shows that the anterior polar cells (a pair of two cells at the pole of the egg chamber) can sense the mechanical properties of the BM. Anterior polar cells' focal adhesion signaling leads to the production of the matrix protease Mmp1, which changes the mechanical properties of the BM from the anterior pole (Fig. 3A) (Ku et al., 2023).

How the BM stiffness is altered at the posterior pole is currently unknown. However, one study showed the requirement of the matrix metalloprotease AdamTS-A for egg chamber elongation; interestingly, this protease was also identified as a target gene of JAK/Stat signaling (Wittes and Schüpbach, 2019).

### A perforated BM in salivary gland branching

The highly dynamic nature of the BM has also been observed during mouse salivary gland development (Harunaga et al., 2014). Here, the authors studied the role of BM remodeling in branching morphogenesis. During the branching of the salivary glands, the BM becomes transiently perforated at the rapidly outgrowing end buds, most prominently at the tip of the buds. Interestingly, in nonexpanding regions, like the cleft and the duct, the BM was uniform and accumulated in this region (Fig. 3B). This structural heterogeneity was observed using the three major constituents of BM: Laminin, Collagen IV, and Perlecan. Live-imaging of Collagen IV shows the local and global dynamics of the BM. A FRAP experiment uncovers the global BM dynamics in this tissue and shines a light on the underlying mechanism. Here, the authors bleached a small region at the bud and used this as a marker to track BM displacement. The BM is moving with a speed of 8 µm/h from the tip of the bud towards the duct region, where it accumulates. Drug treatments reveal a dependency of these BM dynamics on Myosin II contractility and Matrix protease-mediated BM remodeling. This suggests a model in which proteases locally remodel the BM and weaken its mechanical properties, followed by an active pulling of cells on the BM by increased actomyosin contractions and a resulting microperforated BM (Fig. 3B) (Harunaga et al., 2014).

### From the tip: BM dynamics form the VNC

As described above, the shape and condensation of the *Drosophila* VNC depend on the BM. Without proper BM assembly, the VNC fails to condense. Moreover, with Mmp2, Kuzbanian (Adam gene group), and AdamTS-A, a series of matrix-degrading proteases have also been described to be parted in this process (Martinek et al., 2008; Meyer et al., 2014; Pastor-Pareja and Xu, 2011; Urbano et al., 2009). A recent and highly interesting publication used the VNC as a system and demonstrated how *in vivo* assembly of BM components and resulting mechanical heterogeneity leads to organ sculpting (Serna-Morales et al., 2023). Here, unequal incorporation of Collagen IV in the VNC leads to a transient Collagen IV gradient and subsequent mechanical heterogeneity (Fig. 3C). Interestingly, the origin of this gradient has a morphological nature. Hemocytes, the source of Collagen IV in the *Drosophila* embryo, differentiate from a small cell population in the head mesoderm (Holz et al., 2003) and migrate posteriorly to deposit BM components on tissues. As a result, in early embryogenesis, more Collagen IV is located in the anterior than in the posterior region of the VNC. This unequal distribution affects the VNC morpho-dynamics and the shortening of the tissue. Later, when the BM is fully and equally assembled, the VNC undergoes a condensation process (Fig. 3C).

### U-Turn during gonad morphogenesis

During the larval development of *C. elegans*, the characteristic u-shape structure of the gonad has long been thought to be driven by active cell migration of a small group of cells at the tip of the organ, called distal tip cells (DTC) (Sherwood and Plastino, 2018). Hereby, the two-armed gonad first elongates along the ventral side of the worm and then turns back to the center of the animal. Agarwal et al. (2022) uncover now the mechanism of how the BM is differentially remodeled to bring the gonad into its final shape. First, the gonad grows due to the proliferation of the germ cells and elongates in opposite directions. Here, the authors show, using laser ablation experiments, that the BM resists the pressure of the growing organ. If stress is released, the organ rushes forward. Specific expression of Gon-1, an AdamTS metalloprotease, at the tip of the DTS, allows elongation of the organ (Fig. 3D). Hence, the knockdown of this protease leads to the accumulation of Laminin and a disturbed, rounder morphology. FRAP analysis supports these findings since the mobile fraction of Laminin is higher at the tip compared to the proximal region of the gonad. In the second phase, the turning of the gonad, Integrin subunits, and Talin become stronger enriched at the dorsal side to which the cells move. As Integrins are cell-adhesion receptors and Talin is a protein required for integrin activation, this asymmetric protein distribution suggests a BM adhesion-mediated turning of the gonad (Fig. 3D). The authors simulated the effects of adhesion strength and the fraction of time with asymmetric adhesion on gonad morphology to explain the phenotypes of previously observed morphological defects of mutants for Integrins and Talin (Cram et al., 2003; Lee et al., 2001; Meighan and Schwarzbauer, 2007; Wong et al., 2014). Finally, the authors show the requirement of Cdc42, Src, and netrin to regulate Integrin adhesion polarity.

### Anisotropic growth in wing development

The *Drosophila* wing disc is one of the most popular model tissues to study the function of morphogens and cell mechanics for organ growth and shape (Beira and Paro, 2016). The BM has previously been shown to be involved in these processes by mechanically constricting cells (Pastor-Pareja and Xu, 2011, see above), limiting the function of morphogens required for growth (Ma et al., 2017) or sculpting tissues through local BM degradation associated with a decrease of basal tension (Sui et al., 2018). In Harmansa et al. (2023), the authors now show how differential growth between the epithelial layers and the surrounding BM guides the morphogenesis of this organ. The wing disc comprises two monolayered epithelia, the disc proper epithelium (DP) and the peripodial epithelium (PPE), and an overlaying BM around the whole organ. During the main growth phase, the squamous PPE becomes even thinner and the thickness of columnar DP increases, while the entire organ becomes a ‘dome-like’ structure. Now, the authors show a dependence on the BM of these morphological changes. The thickness of the BM at the DP increases. At the same time, the BM on top of the PPE does not change (Fig. 3E). To test whether both regions of the BM are under mechanical stress, photobleaching of a GFP-tagged Col IV with subsequent decellularization was performed. The BM in the DP region became significantly thicker, while the BM at the PPE site was unaffected. This imbalance of the planar growth rates of the DP, PPE, and the BM of the PPE versus planar growth and growth in thickness of DP-BM leads to growth anisotropy, which results in stress and tissue constriction. Furthermore, the authors found a differential expression level of the Matrix metalloprotease 2 (Mmp2). Knockdown of this protease leads to an increased thickness of the BM after decellularization, indicating Mmp2 as the key factor in inducing BM heterogeneity and anisotropic growth.

## Conclusions and future directions

In the past decades, studies on morphogenesis have concentrated on changes in tissue shape driven by a limited repertoire of cell-autonomous activities, such as cell rearrangements, shape changes, and cell migrations (Lecuit and Le Goff, 2007). However, recent studies suggest an important role of extrinsic forces in cellular behavior.

The BM plays a fundamental role in morphogenesis. It assembles in a spatiotemporal fashion, and this leads to a specific composition or structure that is critical to provide biochemical signals or extrinsic forces to the underlying cells when they are required to form tissues and organs. But these complex interactions are poorly understood, and we are just beginning to understand the dynamics of BM components during development and their mechanical contribution in shaping tissues and organs. Furthermore, the BM molecular compositions and the resulting biophysical and biochemical features of different organ BMs are nearly as diverse as their specific biological functions. Along with this, the mechanical role of the BM as an active factor that shapes organs remains unknown in most tissues. Long-term live-imaging and combining genetic manipulation and direct biophysical measurements now allow us to study these underexplored functions.

The material properties of the BM are essential to form and constrict tissues. Stiffness, viscoelasticity, poroelasticity, and maximum strength are different material properties that can influence cell behavior (Chaudhuri et al., 2020; Elosegui-Artola, 2021; Handorf et al., 2015; Janmey et al., 2020). These material properties are properly influenced by characteristics of the BM partially described in this Review: BM composition, turnover, thickness, polarization, density, microstructure, and crosslinking are structural changes described for different mechanisms that can appear during development and direct morphogenesis. Live imaging of fluorescently tagged BM components helped to understand how the BM acted *in vivo* in the past years, and measurements using FRAP or optogenetics can be used to quantify BM turnover and elastic or viscoelastic behavior of BM components (Keeley et al., 2020; Matsubayashi et al., 2017, 2020; Morgner et al., 2023; Soans et al., 2022). Additionally, Atomic force microscopy and Brillouin microscopy have emerged as excellent tools for studying BM mechanics (Prevedel et al., 2019; Töpfer et al., 2022b; Viji Babu and Radmacher, 2019). A combination of visualizing BM dynamics with non-invasive techniques allows the measurement of the biophysical properties of the BM in the living system, which will give huge evidence about the mechanical role of the BM in morphogenesis. A future challenge is to simultaneously investigate the BM dynamics and the underlying mechanical role of the BM in forming tissues and organs.

A problem in matrix biology is the uncoupling of the biochemically and biophysically functions of the BM. As described above, BMs can influence morphogenesis by acting as reservoirs for signaling molecules and providing mechanical cues. Especially, Collagen IV and Perlecan can regulate a series of signaling pathways (Merz et al., 2003; Paralkar et al., 1991; Park et al., 2003; Patel et al., 2007; Wang et al., 2008). Thus, results investigating the mechanical role of BM components for morphogenesis have to be carefully interpreted. Another difficulty comes along with complex protein interactions and dependencies. Studies investigating the role of a single protein in BM function cannot be fully recapitulated without the analysis of the resulting BM composition.

An interesting perspective of matrix biology is understanding why BMs are so diverse between each tissue (Pastor-Pareja, 2020) and the underlying physiological reasons. Why are some components expressed by tissues themselves and others produced in an unspecific manner? Across species, the BM shows diverse structural abilities and differential remodeling. A recent study shows differential gene expression across tissues and suggests an adjustment of BM protein level due to the specific needs of individual tissues (Jayadev et al., 2022). We are now at the starting point of understanding the mechanical role of the BM for morphogenesis and what happens with tissues and organs if it is disturbed.
